# Between traditional remedies and pharmaceutical drugs: prevention and treatment of “Palu” in households in Benin, West Africa

**DOI:** 10.1186/s12889-020-09479-7

**Published:** 2020-09-18

**Authors:** Barikissou Georgia Damien, Carine Baxerres, Edwige Apetoh, Jean-Yves Le Hesran

**Affiliations:** 1 Centre of Training and Research for Population, University of Abomey-Calavi, Cotonou, Benin; 2 UMR261 - MERIT, French National Research Institute for Sustainable Development (IRD), University of Paris Descartes, Paris, France; 3 Centre Norbert Elias EHESS-Campus Marseille La Vieille Charité, 2 Rue de la Charité, 13002 Marseille, France; 4 Ecole doctorale Pierre Louis de santé publique, ED 393 - Santé publique: Epidémiologie et Sciences de l’Information Biomédicale, Paris, France

**Keywords:** Malaria, Herbal medicine, Pharmaceutical medicine, Prevention, Treatment, Benin

## Abstract

**Background:**

In Benin, malaria clinical cases, including the larger popular entity called “Palu” are evoked when people get fever. “Palu” is often self-diagnosed and self-medicated at home. This study aimed to describe the use of herbal medicine, and/or pharmaceutical medicines for prevention and treatment of malaria at home and the factors associated with this usage.

**Methods:**

A cross-sectional survey was conducted in Benin in an urban and in a rural area in 2016. Around 600 households in each place were selected by using a random sampling of houses GPS coordinates of the families. The association between socio demographic characteristics and the use of herbal medicine was tested by using logistic regression models.

**Results:**

In Cotonou (urban), 43.64% of households reported using herbal or pharmaceutical medicine to prevent “Palu”, while they were 53.1% in Lobogo (rural). To treat “Palu” in Cotonou, 5.34% of households reported using herbal medicine exclusively, 33.70% pharmaceutical medicine exclusively and 60.96% reported using both. In Lobogo, 4% reported using herbal medicine exclusively, 6.78% pharmaceutical medicine exclusively and 89.22% reported using both. In Cotonou, the factors “age of respondent”, “participation to a traditional form of savings” and “low socioeconomic level of the household” were associated with the use of herbal medicine.

**Conclusions:**

This study shows the strong use of herbal medicine to prevent “Palu” or even treat it, and in this case it is mostly associated with the use of pharmaceutical medicine. It also highlights the fact that malaria control and care seeking behaviour with herbal medicine remain closely linked to household low-income status but also to cultural behaviour. The interest of this study is mostly educational, with regards to community practices concerning “Palu”, and to the design of adapted behaviour change communication strategies. Finally, there is a need to take into account the traditional habits of populations in malaria control and define a rational and risk-free use of herbal medicine as WHO-recommended.

## Background

A medicine corresponds to “any substance or composition presented as having curative or preventive properties with regard to human or animal diseases” [1]. This definition includes the so-called “modern” medicinal products made by pharmaceutical industries and “traditional” medicines produced locally. According to the World Health Organization (WHO), a traditional medicine is “the total sum of the knowledge, skills, and practices based on the theories, beliefs, and experiences indigenous to different cultures, whether explicable or not, used in the maintenance of health as well as in the prevention, diagnosis, improvement or treatment of physical and mental illness” [2]. In Benin, “traditional” medicines concern mainly herbal medicine (HM). This HM can be used as homemade herbal medicine or manufactured herbal medicine, called “neo-traditional medicine” [3, 4].

In the 2011 World Drug Situation Report, between 70 and 95% of the population in developing countries used HM in combination with pharmaceutical medicines (PM), or separately [5, 6]. The use of HMs is guided by the nature of the disease, the availability and accessibility of PM [7]. In particular, in rural areas, HMs have been identified as the first and sometimes the only solution for health care [8, 9].

After Alma Ata’s 1978 declaration which focused on gross inequalities in the health situation of people, especially in poor income countries [10], access to quality care became a topic of concern to the governments. Many countries adopted the Primary Health Care Strategy, which focuses on equity, community participation, and intersectoral collaboration such as education and health. However, given the financial inability of states to provide the population with a high quality of health care, in 1987, the Bamako Initiative instituted community participation and cost recovery of health care by the people. Unfortunately, this initiative did not achieve its objectives [11]. We observed an increase in disaffection of public health centres and the development of self-medication.

In October 2018, the Astana Declaration [12] reaffirmed the commitments expressed in the Declaration of Alma-Ata of 1978. This declaration supports broadening and extending access to a range of health care services through the use of high-quality, safe, effective and affordable medicines, including, as appropriate, traditional medicines.

Benin is a low-income country located in West Africa. In 2017, around 45% of the people lived in urban areas. Population of children aged under 14 years represented 43.5% of the total population in 2017 [13]. The informal economy is estimated to 55.6% of activities [14]. In 2014, the index of human development was equal to 0.48 [15]. According to the 2017–2018 demographic health survey, the level of education in adults is low: 32% among men and 55% among women never went to school [16]. In 2017, malaria remained a major public health problem in Benin [17], and the first cause of recourse to public health facilities (42%) [18]. In 2016, malaria constituted 42% of all health facility visits and hospitalizations [18]. At the time of the study, pharmaceutical drugs were available in Benin, both in pharmacies and health centres, but also on a highly active informal market.

According to the recommendations of the Ministry of Health (MoH), malaria cases must be confirmed through biological tests before being treated. All the same, Artemisinin-based combination therapies (ACTs) have been recommended since 2004 for the management of uncomplicated malaria attacks [19, 20]. However, as shown in Ouganda by Mpimbaza et al.*,* [21], or Herzt et al.*,* in Tanzania [22], malaria is mainly managed at home through “Palu” diagnosis in Benin [23]. “Palu”, a nosological popular entity [24] in French speaking countries, is associated with fever/chills and various general symptoms such as asthenia, headaches, aches, vomiting, both in adults and children [25, 26]. For a long time, “Palu” was treated as malaria at home and at health centres. Since 2013, Benin health policy introduced a rapid diagnosis test to confirm malaria cases. However, ACT and RDT deployment face stock out issues in some health centres.

Populations develop their own care management behavior. This self-mediation is facilitated by free access to plants in markets but also to PM, both in urban and rural areas.

One of the questions is therefore to determine the weight of herbal therapies in the therapeutic arsenal used by populations. As Peter Bai James showed in a 180-articles review [27], despite the heterogeneity and general low quality of the identified literature, the review highlighted a relatively high use of HM alone or in combination with PM.

In many countries, the search for new treatments is often drawn from population surveys of HM use. This question is not only of interest to Africa. Suswardany et al.*,* [28] showed that in Southeast Asia, traditional plants are widely used, and put forward the same arguments as those found in Africa: lack of accessibility to conventional health services due to geographical and financial barriers, faith in traditional treatments, and the perception of lower severity of malaria symptoms.

Everywhere, health authorities base their management of health problems primarily on the use of pharmaceutical drugs. They give very few and clear recommendations for the use of phytotherapeutic products. However, studies show that many health problems are treated at home by self-medication and, in this context, families can resort to traditional herbal products [9]. This practice delays possible recourse to a health centre and, moreover, if the treatment is not adapted to the health problem, there is a risk of complication. Few studies captured the use of medicines in general populations.

Specifically, this research helps to describe care-seeking behaviour in terms of the use of medicines for children and adults. These practices are not well known because they take place at home and are not recorded statistically. The results of this study should therefore help to assist health authorities by providing data on health practices in the general population and on the use of phytotherapy mainly in case of self-medication. This information is important for health authorities to understand the health-care needs of populations and adjust health policies locally, and for the health care providers who need to communicate with the population and promote officially recommended treatments. It is also an opportunity for biochemists, botanists and public health specialists to take an interest in the active principles of these HMs in order to angle the regional pharmaceutical development.

“Palu”, the most common disease in the Sub-Saharan African countries, is therefore, a particularly pertinent disease to address in order to measure the weight and to understand the exclusive or combined use of PM and/or HM and the factors determining the choice of different kinds of treatments in families.

## Methods

### Study framework

This study was part of the Globalmed research program, implemented in two African countries with different pharmaceutical regulation systems, Benin and Ghana [29]. This research project proposes to study the realities surrounding the global drug market, both in terms of supply (circulation, distribution) and demand (uses, consumption) in the two countries. This article focuses on the data collected in Benin especially on the section on medication used to treat and prevent malaria.

The study was conducted in two areas. Both regions are very different. The urban area of Cotonou, the economic capital of Benin, and the rural area of Lobogo, in the Bopa district, Mono Region, in the South-West of the country. According to the fourth general population census (RGPH4), Cotonou had 679,012 inhabitants in 2013 [30]. Lobogo village had 6000 inhabitants in 2013 [30].

At Lobogo, the rural area, the Community-based Integrated Management of Childhood Illness was implemented. Community health workers ensure the management of uncomplicated malaria cases, diarrhoea and acute respiratory diseases in children by using passive case detection. They are equipped with rapid diagnostic tests, ACT tablets, antibiotics and oral rehydration salts.

In both rural and urban areas, behaviour change communication activities against malaria are conducted towards woman groups and household decision-makers like fathers and grand-parents. In the health centres, ACTs are sold in case of malaria even to children. Malaria cases are often confirmed free of charge. There are sometimes some stock-outs in ACTs or rapid diagnostic tests.

### Study design

Cross-sectional surveys were carried out in April and May 2016 in Cotonou and in November and December 2016 in Lobogo. Households were visited to inquire about the management of health in the family, especially malaria management within adults (above 17 years old) and children under 12 years old.

A household sampling method was used. It was inspired from the methodology used by Shannon et al.*,* [23, 31]. The method used Global Positioning Systems (GPS) coordinates and aerial and satellite photographs. The randomization of GPS points into the study areas was used to select households identified for inclusion in the surveys.

The number of households to include in the studies was calculated using the Schwartz formula [32]. In total, 638 households were visited in Cotonou and 597 households in Lobogo. In the field, households were visited and the head of each household was interviewed to inquire treatments used to treat or prevent “Palu” in the household.

### Data collection

A structured and semi-open questionnaire (Additional file 1) was used to collect the data. The questionnaire was translated by socio-anthropologists into the most local common languages: “Fon” in Cotonou and “Mina” in Lobogo. The questionnaire was administered by enumerators who graduated in socio-anthropology and speak fluently local languages with knowledge of the field studies.

Enumerators were trained for 8 days on the content of the questionnaire and on the technique of the household’s identification. During this period a pre-test of the questionnaire was carried out.

At the start of the survey, the enumerator equipped with a GPS point went to the identified house and interviewed the head of the household. If there were several households, one household was drawn randomly.

The questionnaire was addressed to the head of each household. If the head of the household was absent at the time of the visit, the questionnaire was submitted to his or her spouse or to the adult responsible for the household at the time of our visit.

Data collection took approximately 30 days in each study area. The data was registered with continuous quality control.

### Variables

The questionnaire included sociodemographic items about the respondent such as age; level of education; marital status; religion; nationality; subscription to health insurance policy. Economic characteristics of the households were collected to calculate a wealth index. A set of 17 variables based on the household possession and housing characteristics (nature of habitat, access to resources and property owned by the household etc.) were collected and used to build this index. A multiple component analysis (MCA) was first applied to the modalities of all 17 variables and then a standardized principal component analysis was applied to the main factors resulting from the MCA [33, 34]. By using Garenne’s method [33], the wealth index was categorized into five groups in ascending order of “very poor”, “poor”, “intermediate”, “rich” and “very rich” the highest socioeconomic status.

The head of the household was asked about the types of treatments used to treat or prevent “Palu” by the household members. “Palu” term was used to ask the questions. As said above, in Benin, “Palu” is a “nosological popular entity” [24] associated with fever/chills and various general symptoms such as asthenia, headaches, aches, vomiting, both in adults and children.

Because the same household could use either traditional or pharmaceutical medication or a combination of both types of treatments, the question was asked separately for HM and PM. The same questions were asked about “Palu” prevention.

### Outcome variable

The dependent variable considered for the analysis was the use of PM or/and HM to treat or to prevent a health problem attributed to “Palu”. The use of treatments to cure “Palu” was coded into two modalities: i) families who reported using only PM and ii) families who used both. Very few families, 5% in the urban and 4% in the rural area, reported using only HM and they were excluded from this part of the logistic regression analysis. The “prevention” implies primary prevention by the consumption of HM or PM to avoid the occurrence of “Palu”. The variable “use of treatments to prevent Palu” was binary and coded “yes” if they practice prevention and “no” if not.

### Independent variables

The independent variables used in the regression model are standard and those found in a similar study which determines the associated factors to appropriate medical care-seeking [27, 35]. We assume that these variables have an impact on the ability of people to understand health education programs and practice government recommendations. The variables used to analyze the behavior of the households concerning the treatment or the prevention of “Palu” were age, sex, level of education, religion, place of birth, type of household, level of wealth, health insurance and “tontine”, which corresponds to a membership of local savings and loans activities.

### Statistical analysis

The data was entered into Access 2010 and analyzed using STATA software, version 12 (Stata Corp., TX USA). A descriptive analysis was carried out for the urban and rural areas in order to describe the outcomes of the consumption of treatments (HM and PM). Household characteristics and socio-demographic characteristics of the questionnaire respondents were also described. The associations between the socio-demographic characteristics of the household and the outcomes were tested using a logistic regression model and the top-down step-by-step procedure. The Chi-square or Fisher test, as appropriate, was used to measure the association between two variables. The strength of the associations was estimated using the Odds Ratio (OR) with an estimated 95% confidence interval (CI). The differences were considered statistically significant when the *p*-value was < 5%. The multiple logistic regression analysis was done only for the urban area. Indeed, in the rural area, population and lifestyles (wealth index) are more homogenous than in the city.

## Results

### Descriptive characteristics of the households

In the urban area, a total of 638 households containing 3164 individuals were surveyed. The average age of the questionnaire respondents was 39.5 ± 14.2 years. 66.3% of respondents were women. The average age was 41.0 ± 15.4 years in males and 39.9 ± 13.5 years in females. The average number of individuals per household was 4.96 ± 2.93. 11% of households reported having health insurance and 39% were involved in a “tontine”. According to the wealth index (min 0 and max 17) grouped into five classes, the poorest households represented 5.27% of all the households and the richest households represented 3,91%.

In the rural area, a total of 597 households were surveyed. The average age of the questionnaire respondents was 36.88 ± 14.9. 78.75% of respondents were female. In rural areas, women remain more at home than in urban areas. The average age was 40.7 ± 17.5 years in males and 35.6 ± 13.4 years in females. The average number of individuals per household was 4.42 ± 2.41. 3.7% of households reported having health insurance and 46.55% were involved in a “tontine”. Table 1 describes the socio-demographic characteristics of the respondents and households.

**Table 1:** Characteristics of the respondents in urban and rural areas, Cotonou and Lobogo, 2016

	Study areas			
	Cotonou		Lobogo	
Variables	n	%	n	%
**Age group (years)**				
< 25	62	11.57	103	17.25
[25–35]	158	29.48	197	33.00
[35–45]	136	25.37	143	23.95
[45–55]	85	15.86	73	12.23
[55–65]	56	10.45	29	4.86
> 65	39	7.28	52	8.71
**Sex**				
Male	165	30.78	127	21.27
Female	371	69.22	470	78.73
**School level**				
None	122	22.76	360	60.30
Primary	151	28.17	109	18.26
Secondary	190	35.45	117	19.60
University	73	13.62	11	1.84
**Maternal tongue**				
Fon and related	227	42.35	12	2.01
Goun and related	79	14.74	3	0.50
Mina/Adja and related	125	23.32	578	96.82
North-East and related	13	2.43		0.00
North-West and related	17	3.17	0	0.00
Yoruba	62	11.57	2	0.34
Other	13	2.43	2	0.34
**Place of birth**				
Cotonou	245	45.71	7	1.17
Other	291	54.29	590	98.83
**Religion**				
Catholic	276	51.49	118	19.77
Other Christians	152	28.36	275	46.06
Muslim	82	15.3	2	0.34
Other	26	4.85	202	33.84
**Type of household**				
Monoparental	401	62.85	116	19.43
Biparental	237	37.15	481	80.57
**Profession**				
Agent/employee	135	25.19	26	4.36
Retirement	45	8.4	1	0.17
Trader/retailer	216	40.3	199	33.33
Hand worker	108	20.15	162	27.14
Daily workers	32	5.97	10	1.68
Farmers	0	0.00	199	33.33
**Nationality**				
Beninese	492	91.79	590	98.83
Other	44	8.21	7	1.17

### Use of herbal medicine or pharmaceutical medicine to treat and to prevent “Palu” attacks

#### Treatment of “Palu” attacks

In the urban area, out of 638 households, only 13 respondents reported taking nothing in case of “Palu” (2%) and 82 (12.85%) could not name the treatment usually taken. Out of the 543 respondents who named the treatments taken in the family, 5.34% reported using homemade HM exclusively, 33.70% PM exclusively and 60.96% reported using both (HM + PM).

In the rural area, out of the 575 respondents who named a treatment, 4% reported using HM exclusively, 6.78% PM exclusively and 89.22% reported using HM + PM.

#### Prevention of “Palu” attacks

In the urban area, among the 511 households who answered the questions, 43.64% of individuals said they were using a treatment (HM or PM) to prevent malaria. Among those who named a treatment (*n* = 223), 56.65% used HM exclusively, 23.77% used PM exclusively, and 19.73% used HM + PM.

In the rural area, among the 582 households who answered the questions, 53.1% reported practicing malaria prevention. Among them, 86.75% reported using HM exclusively, 3.31% PM exclusively and 9.93% reported using HM + PM.

### Type of herbal medicine used to prevent or to treat “Palu” attacks

In general, the plants were the same for treatment or prevention. Local names were *Acacia (Acacia nilotica*), Kodo (*Paspalum scrobiculatum*), Moringa (*Moringa Oléifera*), Kinkéliba (*Combretum micranthum*), Tigbé (*Cymbopogon citratus*), Cailcédrat (*Khaya senegalensis*), Kpatiman (*Newbouldia laevis*), and Crincrin (*Corchorus olitorius*). *Artemisia* plants were not cited.

#### Treatment of “Palu” attacks by using herbal medicine

In the urban area, a total of 360 households reported using HM to treat “Palu”, most often (53%) based on several plants (cocktail or mixture). In the rural area, out of 536 households using HM to treat “Palu”, 58.65% declared using only one plant and 41.35% several plants.

#### Prevention of “Palu” attacks by using herbal medicine

In the urban area, 170 households (26.65%) reported using HM to prevent malaria, most often based on a single plant (54.85%), or a cocktail of several plants (45.15%).

In the rural area, 292 households (50.17%) reported using HMs to prevent malaria, most often with a cocktail (64%) or a single-plant (36%).

### Type of pharmaceutical medicine used to prevent or treat “Palu”

#### Treatment of “Palu” attacks

In the urban area, 1118 PMs were taken; indeed, each family could enumerate one or more treatment. According to the declarations: 72.98% were bought in formal structures (pharmacies and health centres), 22.99% in the informal market (fixed or mobile sellers), and 2.23% in both places.

In the rural area, 1128 PMs were taken: 57.89% were bought in formal structures, 37.5% in the informal market, and 5.5% in both places.

People living in the rural area reported using ACTs more often (59.96%) than in the urban area (35.45%), (*p* < 0.0001), whether or not they used HM at the same time. On the contrary, quinine was more heavily used in the urban area (28.79%), compared with rural area (4.49%), (*p* < 0.0001), whether or not associated with HM. Sulfadoxine-pyrimethamine (SP) was cited in the city (7.69%) exclusively. The use of chloroquine was also reported both in urban area (8.79%) and in rural area (9.18%), (Table 2).

**Table 2:** Type of the pharmaceutical medicine cited by the household (PM exclusively group and HM + PM group) used to treat “Palu” at Cotonou and Lobogo, Benin, 2016

Type of pharmaceutical drugs	Cotonou				Lobogo			
	HM + PM Group		PM exclusively Group		HM + PM Group		PM exclusively Group	
	n	%	n	%	n	%	n	%
ACT	117	35.45	89	48.90	307	59.96	22	62.86
Quinine	95	28.79	41	22.53	23	4.49	1	2.86
Chloroquine	29	8.79	7	3.85	47	9.18	2	5.71
Sulfadoxine-pyrimetamine	23	6.97	14	7.69	0	0.00	0	0.00
Apipalu^a^	4	1.21	3	1.65	1	0.20	0	0.00
Paracetamol	35	10.61	21	11.54	99	19.34	4	11.43
Others	13	3.94	5	2.75	4	0.78	0	0.00
Forget the name of the PM	12	3.64	2	1.10	31	6.05	6	17.14
Total	330		182		512		35	

*HM* Herbal Medicine, *PM* Pharmaceutical Medicine, *ACT* Artemisinin-Combined Therapy

^a^Apipalu is a HM therapy anti-malarial medicine, in tablets or syrups, package industrially in Benin, n is the number of drugs cited.

#### Prevention of “Palu” attacks

In the urban area, 97 households declared a total of 204 PM for "Palu" prevention: 72.06% were antimalarial drugs, 17.65% cited paracetamol exclusive and 10.29% of household declared using various drugs including antibiotics, deworming, vitamins and iron. Among the households which declared using antimalarial drugs, 16.18% used ACTs, 20.59% used SP, 19.61% used quinine and 12.25% used chloroquine. It should be noted that seven drugs declared as PM (3.43%) were manufactured herbal medicine against malaria called “Api palu”, the most popular and widely distributed medicine in Benin, even in pharmacies (Table 3)

**Table 3:** Pharmaceutical drugs used to prevent “Palu” in rural and urban area, Benin, 2016

Type of pharmaceutical drugs	Prevention			
	Cotonou		Lobogo	
	n	%	n	%
ACT	33	16.18	17	26.15
Sulfadoxine-pyrimetamine	42	20.59	1	1.54
Quinine	40	19.61	6	9.23
Chloroquine	25	12.25	6	9.23
Apipalu^a^	7	3.43	1	1.54
Paracetamol	36	17.65	17	26.15
Others	21	10.29	17	26.15
Total	204		65	

*ACT* Artemisinin-Combined Therapy

^a^Apipalu is a HM anti-malarial medicine, in tablets or syrups, package industrially in Benin, n is the number of drugs cited

.

In the urban area, according to the declarations, among the 204 drugs used, 81.86% were purchased in formal structures, 15.68% in the informal market, and 1.96% in both places.

In the rural area, among the 65 drugs cited by the 40 households using PM for prevention, 26.15% were ACTs, 9.23% quinine, 9.23% chloroquine and 26.15% paracetamol. The other drugs used (26.15%) were mostly non-steroidal anti-inflammatory drugs (Table 3). Among the drugs cited, 72.31% were bought in formal structures and 27.69% in the informal market.

### Factors associated with the use of herbal medicine to treat “Palu” attacks

When considering the reference group of households reporting consumption of PMs only, the factors associated with the use of HM in combination with PM were age, sex, education, health insurance, tontine, and household wealth. Religion, place of birth, type of household (mono/bi-parent) were not significantly associated (Table 4).

**Table 4:** Association between the use of HM combined with PM to treat “Palu” versus PM exclusively and the respondents and the household’s characteristics, Cotonou, 2016

Variables	PM		PM + HM		Brut OR	CI95%		Adjusted OR^a^	CI		P
	n	%	n	%							
***Respondent information***											
**Age group (years)**											0.0239
< 25	31	17.32	30	9.29	1			1			
(25–35)	57	31.84	88	27.27	1.59	0.87	2.91	1.48	0.78	2.81	0.2242
(35–45)	44	24.58	88	27.24	2.07	1.11	3.84	2.12	1.10	4.09	0.0242
(45–55)	26	14.53	57	17.65	2.26	1.14	4.49	2.37	1.14	4.89	0.0201
(55–65)	10	5.59	37	11.46	3.82	1.62	9.04	3.91	1.59	9.58	0.0029
> = 65	11	6.15	23	7.12	2.16	0.90	5.19	2.72	1.04	7.11	0.0408
** Sex**											
Male	72	39.56	101	30.61	1						
Female	110	60.44	229	69.39	1.48	1.02	2.17				
** School level**											
None	20	11.05	81	24.62	1						
Primary	41	22.65	94	28.57	0.57	0.31	1.04				
Secondary	61	33.70	108	32.83	0.44	0.24	0.78				
University	59	32.60	46	13.98	0.19	0.10	0.36				
** Place of birth**											
Cotonou	89	50.28	145	44.48	1						
Other	88	49.72	181	55.52	1.26	0.87	1.82				
** Religion**											
Catholic	93	51.38	168	51.22	1						
Other Christians	46	25.41	102	31.10	1.23	0.80	1.89				
Muslims	27	14.92	46	14.02	0.94	0.55	1.62				
Others	15	8.29	12	3.66	0.44	0.20	0.99				
***Household information***											
**Type of the households**											
Monoparental	137	75.27	250	75.76	1						
Biparental	45	24.73	80	24.24	0.97	0.64	1.48				
**Health insurance**											
No	151	86.29	294	91.88	1						
Yes	24	13.71	26	8.13	0.56	0.31	0.99				
**“Tontine” group**											
No	115	66.09	175	55.03	1						
Yes	59	31.91	143	44.97	1.59	1.08	2.34				
**Wealth index**											< 0.0001
Very poor	3	1.65	24	7.27	1			1			
Poor	36	19.78	107	32.42	0.37	0.11	1.31	0.42	0.11	1.51	0.1828
Intermediate	73	40.11	143	43.33	0.24	0.07	0.84	0.27	0.08	0.95	0.0425
Rich	55	30.22	51	15.45	0.12	0.03	0.41	0.13	0.03	0.46	0.0019
Very rich	15	8.24	5	1.52	0.04	0.01	0.20	0.04	0.01	0.19	< 0.0001

*HM* Herbal medicine, *PM* Pharmaceutical Medicine

^a^Adjusted OR is provided only for the variables associated to the use of HM + PM at the univariate analysis

The older the household respondent was, the more HM were consumed (trend test, *p* = 0.0029). Conversely, the higher the level of education was, the less the consumption of HM (trend test, *p* < 0.0001). HM was most used in households without health insurance (*p* = 0.0485). On the other hand, the use of HM was higher in households membership of a “tontine” (*p* = 0.0171). Finally, households in the highest wealth used less HM (trend test, *p* < 0.0001), (Table 4).

In the multiple logistic regressions, the variables significantly associated with HM use were the “age of respondent”, and the “wealth index” (Table 4).

### Factors associated to “Palu” attacks prevention

In the univariate analysis, considering households which do not practice malaria prevention as reference group, the respondent’s level of education (*p* = 0.0465), household type (mono/biparentality) (*p* = 0.0031) and “wealth index” (*p* < 0.0001) were significantly associated with prevention practices (Table 5). The prevention decreased as the level of education increased. Two-parent households had a higher practice of malaria prevention, OR = 1.23 [1.23–2.80]. There was no significant difference with “age of respondent”, “gender”, “religion”, “wealth level”, “health insurance” and “tontine group”.

**Table 5:** Association between the respondents and the household’s characteristics and “Palu” prevention by the HM or PM, Cotonou, 2016

Variables	No		Yes		OR brut	IC95%		*p*
	n	%	n	%				
***Respondent information***								
**Age group (years)**								0.0615
< 25	42	14.89	22	10.05	1			
(25–35)	94	33.33	63	28.77	1.32	0.71	2.44	0.4259
(35–45)	66	23.4	57	26.03	1.69	0.89	3.19	0.1174
(45–55)	33	11.7	39	17.81	2.31	1.14	4.68	0.0215
(55–65)	31	10.99	17	7.76	1.17	0.53	2.60	0.9089
> = 65	16	5.67	21	9.59	2.80	1.20	6.52	0.0301
**Sex**								
Male	98	34.75	72	32.88	1			
Female	184	65.25	147	67.12	1.09	0.74	1.59	0.6602
**School level**								0.0465
None	48	17.14	58	26.48	1			
Primary	75	26.79	58	26.48	0.64	0.38	1.07	0.0885
Secondary	97	34.64	66	30.14	0.56	0.34	0.92	0.0227
University	60	21.43	37	16.89	0.51	0.29	0.89	0.0187
**Religion**								0.5703
Catholic	138	49.46	120	54.79	1			
Other Christians	80	28.67	60	27.4	0.86	0.57	1.30	0.4844
Muslims	44	15.77	30	13.7	0.78	0.46	1.33	0.3635
Others	17	6.09	9	4.11	0.60	0.26	1.42	0.2493
**Place of birth**								
Cotonou	124	44.13	100	46.95	1			
Other	157	55.87	113	53.05	0.9	0.62	1.28	0.5330
***Household information***								
**Type of the households**								
Monoparental	227	80.5	151	68.95	1			
Biparental	55	19.5	68	31.05	1.89	1.23	2.80	0.0031
**Health insurance**								
No	243	87.41	192	89.30	1			
Yes	35	12.59	23	10.70	0.83	0.48	1.46	0.5183
**“Tontine” group**								
No	171	62.87	129	60.28	1			
Yes	101	37.13	85	39.72	1.12	0.77	1.61	0.5602
**Wealth index**								< 0.0001
Very poor	3	1.65	24	27.7	1			
Poor	36	19.78	107	29.58	0.37	0.11	1.30	0.1230
Intermediate	73	40.11	143	30.99	0.24	0.07	0.84	0.0253
Rich	55	30.22	51	11.74	0.12	0.03	0.41	0.0008
Very rich	15	8.24	5	1.52	0.04	0.01	0.20	< 0.0001

Although age was not associated with prevention practice (*p* = 0.0615), when considering the age group (< 25 years) as reference, a higher prevention practice was noted in the age groups (45–55), (*p* = 0.0215) and > 65 (*p* = 0.0301).

In the multiple logistic regressions, only the variable “type of household” remained significantly associated with “Palu” prevention (Table 5).

## Discussion

The objective of the study was to analyze the practices of “Palu” management in Beninese households, in terms of both treatment and prevention. The analysis focused specifically on the use of HM and PM in both rural and urban areas. The study also highlighted the factors associated to the use of HM with or without PM in the urban area. The main result of the study shows that populations widely use HM in addition with pharmaceutical treatments which are commonly used in households to manage health problems.

### Use of herbal medicine therapy

While households use mainly PM (> 90%) to treat “Palu”, a very high proportion of them, 61% in urban area and 89% in rural area, also reported using HM.

In 2011, Abdullahi’s review of articles on trends and challenges in traditional medicine in Africa suggested that the use of HM would be higher in countries with low availability/accessibility or poor quality of health services [36]. These data were confirmed by WHO investigation and analysis [9]. Their use would allow populations to reduce direct and indirect expenses they would have to incur if they had to go to a health centre [37, 38]. Nevertheless, the notion that HM use in Africa is most commonly associated with lower access to conventional medical resources is rather simplistic. Indeed, according to our results, a recent review of traditional medicine in Sub-Saharan Africa has shown that even in the presence of PM, HM use remains high [27].

This phenomenon is not limited to developing countries. In northern countries, where the health system is considered efficient, the use of HM is also high, especially in Japan where about 72% of Japanese physicians prescribe some HMs to their patients [39, 40]. Similarly, in France, a survey conducted in 2008 showed that 20% of patients seen in consultation took HM in parallel with a pharmaceutical treatment. However, it is noted that herbal products are considered as “alternative medicine”, but they are rarely spontaneously reported by patients to the physician [41]. This use of HM all over the world suggests that the use of plants to treat or prevent diseases is also due to cultural and historical factors [42].

The use of HM should be stronger in rural areas where the conditions of availability, proximity and quality of plants are high and health coverage by the public health system is low [9, 36, 43].

A large part of households declared using both PM and HM. However, we did not ask to the respondents whether these were combinations of treatments or whether they were used one after the other. Numerous studies in Africa have shown this combined use of HM and PM in cases of self-medication, even after consultation at health centres [42, 44, 45]. A qualitative study carried out in Burkina Faso described different practices. According to this study, all scenarios are possible - PM or HM - first or jointly, but also HM even if there was a consultation at a health centre [45]. Another study that took into account 25 cases of severe malaria in rural children in Mali showed that HM are almost always used as first-line treatment at home. However, in 3/4 of the cases, the use of PM (with HM or alone) was also reported [46]. PM is likely to be used when symptoms persist despite the use of HM. More generally, treatment choices and treatment sequences may change depending on disease course, persistence or aggravation of symptoms and history of severe access in the household [46, 47].

This practice of self-medication based on traditional practices is important because of the high costs of modern health but also for all the knowledge it represents and the related care possibilities. This approach by the HMs constitutes an opportunity for the implementation of WHO’s recommendation of encouraging self-care. WHO has defined “self-care” as “the ability of individuals, families and communities to promote health, prevent disease, maintain health, and to cope with illness and disability with or without the support of a health-care provider” [48, 49]. In this document, the authors propose an overview of common traditional practices in self-care used for promoting health, preventing illness, and managing diseases.

Moreover, they report that self-care will ease the burden of the overstretched health systems, and could be taken in account in universal coverage. However, this approach requires precautions: some diseases such as malaria have the particularity of a risk of rapid worsening if treatment is not adapted. In malaria endemic countries, it is recommended to test the blood for malaria diagnosis in case of fever. It is also recommended to take complete treatment, as advised by the hospital/health centre.

We observed the use of diversified anti-malarial treatments including ACT and quinine, and sometimes inadequate antimalarial drugs like SP or chloroquine. The use of drugs no longer recognized as effective was already found in Benin and in other countries [50, 51]. The question is to know if the great diversity of available antimalarial drugs and the multiplicity of information sources (caregivers, neighbors, and media), mostly in urban areas, pushes people to follow their own ideas rather than official recommendations. In addition, in urban areas, there is a large offer of health care services from the private sector. These health care services do not have access to subsided less expensive ACTs and related trainings, while in rural areas the community health workers can only prescribe ACTs [52, 53].

### Prevent “Palu” attacks

Almost 19% of households in the urban area and 7% in the rural area also reported using PM to prevent malaria. However, no malaria prevention is recommended by the MoH (except for pregnant women). In fact, these treatments are indeed a prevention of diseases and symptoms, rather than a “health maintenance”. Almost 1/5 of the people declared that they did not know whether the treatment they usually took was against “Palu” or not. In Benin and in other countries, there is a strong culture of “well-being maintenance”, “health maintenance”, most often through the use of HM or PM [25]. Thus, it is common for people to take herbal teas, vitamins, analgesics on a daily basis to maintain health. The culture of health maintenance corresponds to a “popular and universal” prevention against all the little daily health problems that families have to deal with and “Palu” could be considered also as common pathology, well known by populations. Moreover, people perceive different degrees of severity associated with “Palu” and gender when they speak about “male palu” and “female palu” [54].

These widespread prevention practices are rooted in popular perceptions of health maintenance with HM [25]. The distinction between curative, preventive and health-promoting care is not always easy in practice. PM or HM are used for example to “strengthen the blood” and “maintain the belly clean”. These preventive practices are used to strengthen the body of the individual overall, to make them less vulnerable to diseases.

The consumption of deworming drugs, for example, widely promoted by health professionals, is perceived both as curative (eliminate the dirt present in the belly) and preventive (this dirt is likely to trigger diseases, such as “Palu”). Mothers who administer drugs to one of their children who are ill often take advantage to also give this treatment, preventively, to the rest of the siblings. In the same way, it is sometimes necessary to prevent, by the consumption of medicine, a disease that several signs already announce, even if they do not necessarily constitute symptoms in the biomedical sense.

“Herbal teas”, based on specific leaves, roots or barks, can be used to treat diseases like “Palu”, “measles”, “fatigue”, or symptoms (stomach pain, toothache, fever, body aches, coughing). But they are mainly consumed in disease prevention and more generally to maintain good health.

More and more often in urban areas, populations use PM to prevent “Palu”. This use of PM is probably due to several reasons: 1) knowledge of effective drugs to treat malaria and in people’s mind their possible effectiveness in preventing it, 2) within older people, the memory of malaria weekly chemoprophylaxis with chloroquine [55] and finally 3) IPT, prevention by SP given to pregnant women three times during pregnancy. Therefore, we can postulate that populations, with a culture of health maintenance and the will to avoid malaria, are creating their own rules of prevention and treatment of “Palu”.

### Use of ACT and quinine

Although ACT and quinine were the drugs recommended by the MoH, families reported using an ACT with or without an HM only in about 40% of cases in urban areas and 60% of cases in rural areas. Surprisingly, it was in urban areas, where information and drug distribution is normally most active, that a lower rate of reported use of ACTs was observed. In Benin’s according to malaria control policy, free access to ACT is only possible in rural areas. This may easily explain the greater use of ACTs in rural areas compared to urban areas. Other hypotheses may explain this result. The belief that the efficacy of ACTs is declining seems to be widespread and is sometimes relayed by health workers who attribute treatment failures, sometimes simply due to a positive rapid diagnosis test (asymptomatic parasitaemia) during the course of any viral infection or in the event of incomplete adherence to a treatment. In a study conducted in Burkina Faso, parasite resistance to antimalarial drugs was cited by prescribers as a reason for non-compliance with the national protocol for uncomplicated malaria cases [56]. In addition, and as a coincidence, it is noted that in urban areas there was a strong declaration of quinine use in contrast to rural areas where this use was low. To reinforce this hypothesis, following the law of supply and demand, a recent study conducted in Benin showed that quinine is one of the most available drugs among private input providers [52].

The health system is therefore faced with a therapeutic wandering of the population who will orient themselves according to diverse and sometimes contradictory information towards what seems to them to be the safest molecule and quinine (in spite of the known side effects) historically and practically meets the needs of the population.

The greater use of PMs to treat malaria in cities does not therefore translate into better compliance with the malaria control recommendations in the country. There is also a use of drugs that are no longer recommended (SP) or even banned from the market (chloroquine). These practices deserve health authorities’ attention.

### Integrated use of HM and PM for diseases management

Whatever the context, low-income countries populations continue to use HM. Governments and the international community are sensitive to it. According to the National Malaria Control Program policies, the health workers need supportive supervision and a strengthened healthcare system with reliable access to essential medicines [57, 58]. Improved monitoring to address issues immediately, such as stock-outs, will lead to improved confidence in the program and in consistent behaviour of visiting a CHW or hospital at the onset of fever or illness. Finally, there is a need to insure the capacity building of BCC officers of health intervention projects in order to improve the quality of social intervention at the community level. As in other low-income countries without universal health insurance, Benin’s population faces lack of cash for care seeking at the health centre. There is a need to improve access to care in Benin through financial protection and community-based services.

According to the traditional medicine strategy (2014 to 2023) [2], WHO will continue to provide policy guidance to Member States on how to integrate traditional and complementary medicine services within their national and/or subnational health care systems, as well as the technical guidance that would ensure the safety [59], quality, and effectiveness of such traditional and complementary medicine services.

The governments also adhere to this opportunity. For example, in the MoH of Benin, a National Program of Traditional Pharmacopoeia and Medicine (NPTPM) has been established with a view to improve the coverage of the health needs of the Beninese population through effective and efficient use of Pharmacopoeia. The objectives of the NPTPM are to promote and integrate traditional pharmacopoeia and medicine into the national health system in Benin. In 2016, the Beninese government decided to set up a committee to draft a project document for the installation of a reference research and innovation laboratory for herbal medicines in the country [60]. In all cases, it is important to frame the simultaneous use of MH and PM by information and education programs. However, the different drug markets (public, private and informal) make the official message to be delivered to the population complex. Moreover, the issue of stock outs of antimalarial drugs at health centres is a crucial issue that hinders a deep evaluation of behavior in drug consumption. When the patients or caregivers know that the health worker does not have ACT to treat himself or his/her child, their incentive to go to the health worker is greatly decreased, which is a potential key reason to use mixed treatments.

Finally, use of HM in the context of WHO self-care seeking requires precautions especially in case of suspicion of malaria in the context of poor health services offer. If low-income countries improve the management of their health systems, HMs could be offered to patients as in developed countries, either for their effectiveness if it has been proven or as a complementary treatment.

More generally, the use of medicines (HM or PM) could refer also to a sociological process. Then it is legitimate to have a debate on the opportunity of self-care [49] and about the place of traditional (and neo-traditional) medicine in the therapeutic arsenal.

## Study limitation

This study measured the weight of “Palu” in the population by investigating the type and quantity of drugs used to prevent and treat it. However, we did not confirm that “Palu” episodes were malaria cases by using malaria tests.

Biases are inherent in cross-sectional surveys. In this study, we assume that respondents could not report all treatments usually given to prevent or treat malaria. Concerning wealth indicator, the majority of the population works in the informal sector and parts of the wealth indicator were self-reported.

## Conclusion

In Benin, authorities recommend the use of ACT to treat malaria. However, States also advocate the use of traditional pharmacopoeia and set up structures to manage the relations with traditional practitioners. At the same time, families practice self-medication a lot to treat malaria. They use PM mainly associated with HM. HM is also used to prevent malaria. This strong use of HM is observed everywhere even if these practices are less used in urban areas.

In the obligation to cover their own health costs, the families build their own treatment regimes, mixing HM and PM, facilitated by free access to a wide range of PM in the pharmacies or in the informal markets.

The use of HMs as a complement to the use of PMs is proposed by states and WHO. In the case of malaria control, this can only be done under very strict control by health authorities, who must first validate their use: efficacy, potential adverse reactions and interactions with existing pharmaceutical medicines. If low-income countries improve the management of their health systems, HMs could be offered to patients as in developed countries, either for their effectiveness or as a complementary treatment. These data could contribute to a scientific and academic debate on the use of HM and PM but also to some sort of community outreach and education that could help to improve practices surrounding both herbal and pharmaceutical medicine use.

## Supplementary information

**Additional file 1.** Questionnaire WP3: Access to medicines in general population GLOBALMED project.

## Data Availability

“The datasets used and/or analysed during the current study available from the corresponding author on reasonable request.”

## Abbreviations

ACT: Artemesinin-based Combination Therapy
GPS: Global Positioning Systems
HM: Herbal medicines
MCA: Multiple component analysis
MoH: Ministry of Health
NPTPM: National Program of Traditional Pharmacopoeia and Medicine
PM: Pharmaceutical medicines
RGPH4: Fourth general population census
SP: Sulfadoxine-pyrimethamine
WHO: World Health Organization
