# Supplementary material for: Between traditional remedies and pharmaceutical drugs: prevention and treatment of “Palu” in households in Benin, West Africa
Source: BMC Public Health. 2020 Sep 18;20:1425. doi: 10.1186/s12889-020-09479-7 (PMC7501613; doi:10.1186/s12889-020-09479-7)
Supplement: Supplementary file 1 — Additional file 1. Questionnaire WP3: Access to medicines in general population GLOBALMED project. [file 12889_2020_9479_MOESM1_ESM.docx]

Additional files1

GLOBALMED - Questionnaire WP3: Access to medicines in general population

Name of Location: _ _ _ _ _ _ _ _ _ _ _ _ _ _ _ _ _ _ _ _ _ _ _ _ _ _ _ _ _ _ _ _ _ _ _ GPS point number: |__|__|__|__|

House investigated: ☐ Choice 1 ☐ Choice 2 ☐ Choice 3 ☐ Choice 4 ☐ Choice 5 ☐ Choice 6

**General informations**

Initial of interviewer: |__|__| Date of the interview: |__|__|/|__|__|/|__|__|__|__| Number of households in the place: |__|__|__|

***Draw a household by random if several***

**A. Sociodemographic data of the household**

Start time of interview*: |__|__|h/|__|__| min*

**A1)** How long have you live in Cotonou/Lobogo? _ _ _ _ _ _ _ _ _ _ _ _ _ _ _ _ _ _ _ _ _ _ _ _ _ _ _ _ _ _ _ _ _ _ _ _ _ _ _ _ _ _ _ _ _ _ _ _ _ _

**A2)** Collect data in this table

|  | **Age** | **Sex** | **School level** | **Main activity** | Marital status ***** | **Religion*** | **Nationality** | **Ethnicity** | **Place of birth** |
| --- | --- | --- | --- | --- | --- | --- | --- | --- | --- |
| **Respondent** (s) |  |  |  |  |  |  |  |  |  |
| ☐ **HH**  **Other**  ☐ **spouse**  ☐**other specify……………** |  | ☐F ☐M |  |  |  |  |  |  |  |
| Partner |  | ☐F ☐M |  |  |  |  |  |  |  |

**A3) Does the** partner live in the household? ☐ Yes ☐ No ☐ Other, specify _ _ _ _ _ _ _ _ _ _ _ _ _ _ _ _ _ _ _ _ _ _ _ _ _ _

**A4)** How many adults aged 18 and above live in the household, including yourself? |__|__|__|, of whom |__|__|__| present during the investigator's visit

**A5)** How many children aged 12-17, 6-11, 0-5 years live in the household? 12-17 yrs. |__|__| A**6) 6**-11yrs __|__| **A7)** 0-5 yrs. __|__|

**A8)** In the household, do you have:

| **Electricity** | **Television** | **Fridge/Freezer** | **Generator** | **Gas Cooker** | **Telephone** | Sanitary facilities |
| --- | --- | --- | --- | --- | --- | --- |
| ☐ Yes ☐ No | ☐ Yes ☐ No | ☐ Yes ☐ No | ☐ Yes ☐ No | ☐ Yes ☐ No | ☐ Yes ☐ No | ☐Water Closet W.C ☐KVIP  ☐ Public toilet ☐Pit latrine  Others: _ _ _ _ _ _ _ _ _ _ __ _ _ _ _ |

**A9)** Fill in the table by asking questions

| **Questions** | **Responses** |
| --- | --- |
| **A9a)** What is the main source of water you drink in the household? \|__\|__\|  Other, specify: ________________________ | ***Running Water 7=****Rain water*  *1=household tap 8=Truck Water*  *2=Common faucet*  *3=public tap/pipe borne water* ***Surface water***  *4 Borehole/ pump 9= water from River*  *10=* dam / lake / pond /irrigation canal  ***Water from the well***  *5=covered well 11=* water in sachet  *6=no covered well 12=Bottle water* |
| **A9b)** What is the main source of water you use to cook, wash dishes or to wash your hands in the household? \|__\|__\|  Other, specify: ________________________ |  |

**A10) nature of the habitat**: |__| *(1=Individual house; 2=Apartment; 3=Compound house);* Other, specify: _ _ _ _ _ _ _ _ _ _ _ _ _ _ _ _ _ _ _ _ _ _ _ _ _ _ _ _ _

**A11)** Nature of the floor of the housing: |__| (*(1=Cemented; 2=Tiled/Terazzo; 3=Sandy; 4=Clay)*; Other, specify: _ _ _ _ _ _ _ _ _ _ _ _ _ _ _ _ _ _ _ _ _ _ __ _ _ _ _ _

**A12)** Nature of the walls of the housing: |__| *(1=Cemented; 2=Plywood; 3= Clay);* Other, specify: _ _ _ _ _ _ _ _ _ _ _ _ _ _ _ _ _ _ _ _ _ _ _ _ _

**A13)** Nature of the roof of the housing: |__| *(1=zinc plate; 2=Roof Tiles; 3=Straws; 4=cement roof);* Other, specify: _ _ _ _ _ _ _ _ _ _ _ _ _ _ _ _ _ _ _ _ _ _ _ _ _ _ _

**A14)** What is your status in the housing: |__| *(1=Owner; 2=Family Owned; 3=Tenant)*; Other, specify: _ _ _ _ _ _ _ _ _ _ _ _ _ _ _ _ _ _ _ _

**A15)** Do you own another house, apartment, or land? ☐ Yes ☐ No; Other, specify: _ _ _ _ _ _ _ _ _ _ _ _ _ _ _

**A16a)** Are there any other households living in the compound? ☐ Yes ☐ No ☐Don’t know ☐ Refusal

**If Yes, go to the next question. If No go to question B1**

**A16b)** *If yes.* How many households? |__|__|__|

**A16c)** Are you related to some households? ☐ Yes ☐ No **A16d)(*If Yes*)** How many households are you related? |__|__|

**A16e)** What are your family ties to these households? |__|__| **;**  |__|__| **;** |__­|__| **;**  |__|__| **;**

☐ Parent ☐ Siblings ☐Extended family ☐ Other Relations

**B.** Pharmaceutical and traditional treatments used for Malaria in the household

**B1)** Which **traditional remedies** do you often use to **treat malaria** in the household? ☐ Nothing ☐ Don’t know ☐ Refusal

| Name of remedy | Composition of the remedy ***** | Forms of Preparation ***** | Place of supply ***** |
| --- | --- | --- | --- |
|  |  |  |  |
|  |  |  |  |
|  |  |  |  |
|  |  |  |  |

**B2)** Which **traditional remedies** do you often use to **prevent malaria** in the household? ☐ Nothing ☐ Don’t know ☐ Refusal

| Name of remedy | Composition of the remedy ***** | Forms of Preparation ***** | Place of supply ***** |
| --- | --- | --- | --- |
|  |  |  |  |
|  |  |  |  |
|  |  |  |  |
|  |  |  |  |

**B3)** Which **pharmaceutical medicine** do you often use to **treat malaria** in the household? ☐ Nothing ☐ Don’t know ☐ Refusal

| Name | Form of Medication***** | Place of Purchase ***** |
| --- | --- | --- |
|  |  |  |
|  |  |  |
|  |  |  |
|  |  |  |

**B4)** Which pharmaceutical medicine do you use to prevent malaria in the household? ☐ Nothing ☐ Don’t know ☐ Refusal

| Name ***** | Form of Medication ***** | Place of supply ***** |
| --- | --- | --- |
|  |  |  |
|  |  |  |
|  |  |  |
|  |  |  |

**B.** Pharmaceutical and traditional treatments used for Chronic Disease in the household

**B5a)** In the **household,** are there people who suffer from a disease for which they take drugs all the time? ☐ Yes ☐ No ☐ Don’t know ☐ Refusal

***If not, go to the question B6a***

**B5b**) If so, can you give me information on the pharmaceutical drugs or traditional remedies used? ☐refusal

| *1=Adult*  *2=Child* | **Disease name** | **Medicine Name** | **Type of treatment*** | **Form of Medication*** | **Place of supply** | **Diagnosis made by a medical doctor?** |
| --- | --- | --- | --- | --- | --- | --- |
|  |  |  |  |  |  | ☐ Yes ☐ No ☐ Don’t know ☐ Refusal |
|  |  |  |  |  |  | ☐ Yes ☐ No ☐ Don’t know ☐ Refusal |
|  |  |  |  |  |  | ☐ Yes ☐ No ☐ Don’t know ☐ Refusal |
|  |  |  |  |  |  | ☐ Yes ☐ No ☐ Don’t know ☐ Refusal |
|  |  |  |  |  |  | ☐ Yes ☐ No ☐ Don’t know ☐ Refusal |

**B. Pharmaceutical and traditional treatments used for Other Disease in the household**

**B6a) Apart from** malaria and chronic diseases, are there members of the household who use **traditional remedies**? ☐ Yes ☐ No ☐ Don’t know ☐ Refusal

***If not, go to the question B7***

**B6b)** If so, what are the name of these remedies, their presentations and why are they used?

| *1=Adult*  *2=Child* | **Name of remedy** | **Composition of remedy*** | **Forms of Preparation *** | **(Symptom/ Disease) Reason for taking the treatment *** | **Place of supply*** |
| --- | --- | --- | --- | --- | --- |
|  |  |  |  |  |  |
|  |  |  |  |  |  |
|  |  |  |  |  |  |
|  |  |  |  |  |  |

**B7)** Apart from malaria and chronic diseases, what are the **main health problems** that occurs in the household? Can you tell me the **pharmaceutical drugs** used? (*Health problem= all symptoms and diseases).*  ☐ Nothing ☐ Don’t know ☐ Refusal

| *1=Adult*  *2=Child* | **Health problem** | **What causes** | **Treatment name** | **Form of Medication*** | **Place of supply *** |
| --- | --- | --- | --- | --- | --- |
|  |  |  |  |  |  |
|  |  |  |  |  |  |
|  |  |  |  |  |  |
|  |  |  |  |  |  |
|  |  |  |  |  |  |

**B8a)** In the household, how many people had a **health event** in the last 7 days prior the survey? |__|__|among whom |__|__| 0-11years old

**B8b)** In the household, how many **people had malaria** in the last 7 days preceding the survey? |__|__|among whom |__|__| 6-11years et |__|__| 0-5 years old

**C.** Episode of malaria in the household

**C1)** When was the last time any adult member of the household had “malaria”? _ _ _ _ _ _ _ _ _ _ _ _ _ _ _ _ _ _ _ _ _ _ _ _ _ _ _ _ _ _ _ _ _ _ _ _ _ _ _ _ _ _ _

**If there is any adult C1a) Age** |__|__| C1b) Sex |__|

**C2)** Which symptoms make you think he/she had malaria *(****Do not quote***)? ☐ Don’t know ☐ Refusal

☐ Fever ☐ Weakness ☐ Cough ☐Vomiting ☐ Headache ☐ Body pain

☐ Cold/Chill ☐ Dizziness ☐ Yellow urine ☐ Diarrhea ☐ Loss of appetite ☐ Bitter taste

☐ Tiredness ☐ Others, Specify _ _ _ _ _ _ _ _ _ _ _ _ _ _ _ _ _ _ _ _ _ _ _ _ _ _ _ _ _ _ _ _ _ _ _ _ _ _ _ _ _ _ _ _ _ _ _ _ _ _ _ _ _ _ _ _ _ _ _ _ _ _ _

**C3)** What is the first thing the person did when he thought about malaria **(Action*)***?* |__| ☐ Other, Specify _ _ _ _ _ _ _ _ _ _ _ _ _ _ _ _ _ _ _ _ _ _ _ _ _ _ _ _ _ _.

***If C3 = "*** Visit to a health professional ***“, go* to the next question (C4a). If no, go to *question C5a***

**C4a*)*** Did the health worker asked for an analysis to confirm that it is malaria? ☐ Yes ☐ No ☐ Don’t know ☐ Refusal

**C4b)** Did he take anything before going to the health professional? ☐ Yes ☐ No ☐ Don’t know ☐ Refusal

**C4c)** *If Yes.* What did he take? Name of treatments _ _ _ _ _ _ _ _ _ _ _ _ _ _ _ _ _ _ _ _ _ _ _ _ _ _ _ _ _ _ _ _ _ _ _ _ _ _ _ _ _ __ _ _; ☐ Don’t know ☐ Refusal

**C4d)** Which treatments have been prescribed by the health professional? ☐ Don’t know ☐ Refusal

| **Name of treatment** | **Form of Mediction *** | **Place of supply** |
| --- | --- | --- |
|  |  |  |
|  |  |  |
|  |  |  |
|  |  |  |
|  |  |  |
|  |  |  |

If you have just completed C4a, C4b, C4c, C4d, go to question C6.

**C5a)** If he did not consult any health professional, what did he take to treat malaria?

| **Name of treatment** | **Type of treatment*** | **Form of Medication *** | **Dosage** | **Place of supply** | **Price** | **advised By *** | **Self-medication** | **Outcome*** | ***Action* 2*** |
| --- | --- | --- | --- | --- | --- | --- | --- | --- | --- |
|  |  |  |  |  |  |  |  |  |  |
|  |  |  |  |  |  |  |  |  |  |
|  |  |  |  |  |  |  |  |  |  |
|  |  |  |  |  |  |  |  |  |  |

**C5b)** Did he make a blood test to confirm malaria? ☐ Yes ☐ No ☐ Don’t know ☐ Refusal

**C6)** Ministry of Health recommends combinations of artemisinin (ACT) for malaria treatment; Do you know ACT? ☐ Yes ☐ No

**C7)** Do you know:

| **Coartem** | **Artefan** | **Combiart** | **Danmether** | **Lonart** | **Ipca** | **Lumartem** | **P-Alaxin** | **Malar-2** |
| --- | --- | --- | --- | --- | --- | --- | --- | --- |
| ☐ Yes ☐ No | ☐ Yes ☐ No | ☐ Yes ☐ No | ☐ Yes ☐ No | ☐ Yes ☐ No | ☐ Yes ☐ No | ☐ Yes ☐ No | ☐ Yes ☐ No | ☐ Yes ☐ No |

***If the household contains at least one child aged 0-11, go to the next question (C8). If not, go to Part D***

**C8)** In the household, when was the last time a child had malaria? _ _ _ _ _ _ _ _ _ _ _ _ _ _ _ _ _ _ _

**C9)** Which symptoms suggested that it was malaria?

☐ Fever ☐ Weakness ☐ Cough ☐Vomiting ☐Headache ☐ Body pain

☐ Cold/Chill ☐ Dizziness ☐ Yellow urine ☐ Diarrhea ☐ Loss of appetite ☐ Bitter taste

☐ Tiredness ☐ Others, specify _ _ _ _ _ _ _ _ _ _ _ _ _ _ _ _ _ _ _ _ _ _ _ _ _ _ _ _ _ _ _ _ _ _ _ _ _ _ _ _ _ _ _ _ _ _ _ _ _ _ _ _ _ _ _ _ _ _ _ _ _ _

**C10)** What is the first thing the person did when he thought about malaria **(Action*)***?* |__| ☐ Other, Specify _ _ _ _ _ _ _ _ _ _ _ _ _ _ _ _ _ _ _ _ _ _ _ _ _ _ _ _ _ _.

***If C10 = "Visit a health professional", go to the next question (C11a). If no, go to question C12a***

**C11a*)*** Did the health professional asked him (or her) for blood test to confirm malaria?☐ Yes ☐ No ☐ Don’t know ☐ Refusal

**C11b)** Was he been given anything before taking him to the health professional? ☐ Yes ☐ No ☐ Don’t know ☐ Refusal

**C11c)** *if yes.* What did they give him? Name of treatments _ _ _ _ _ _ _ _ _ _ _ _ _ _ _ _ _ _ _ _ _ _ _ _ _ _ _ _ _ _ _ _ _ _ _ _ _ _ _ _ _; ☐ Don’t know ☐ Refusal

**C11d)** What medications have been prescribed by the health professional? ☐ Don’t know ☐ Refusal

| **Name of treatment** | **Form of Medication *** | **Place of supply** | **In your opinion, against which symptom was this medication prescribed for?** |
| --- | --- | --- | --- |
|  |  |  |  |
|  |  |  |  |
|  |  |  |  |
|  |  |  |  |
|  |  |  |  |

**C12a)** If the child was not taken to a health professional, what was given to him to treat malaria?

| **Name of treatment** | **Type of treatment*** | **Form of Medication*** | **Dosage** | **Place of supply** | **Price** | **Advised By *** | **Self-medication** | **Outcome*** | ***Action* 2*** |
| --- | --- | --- | --- | --- | --- | --- | --- | --- | --- |
|  |  |  |  |  |  |  |  |  |  |
|  |  |  |  |  |  |  |  |  |  |
|  |  |  |  |  |  |  |  |  |  |
|  |  |  |  |  |  |  |  |  |  |

**C12b)** Did he make blood test to confirm malaria? ☐ Yes ☐ No ☐ Don’t know ☐ Refusal

**D.** Knowledge and lifestyle

**D1) Main means of transport**

| **Questions** |  | How much does travel cost?**?** |
| --- | --- | --- |
| **D1a)** In the household, what is your main means of transport to go to the health facility? | ☐by foot ☐Bike ☐Personal car ☐ Public transport ☐Other, Specify______________ |  |
| **D1b)** In the household, what is your main means of moving to the nearest pharmacy? | ☐by foot ☐Bike ☐Personal car ☐ Public transport ☐Other, Specify______________ |  |
| **D1c)** In the household what is your main means of transport to the nearest drug Sellers? | ☐by foot ☐Bike ☐Personal car ☐ Public transport ☐Other, Specify______________ |  |

**D3)** Did you sleep under a mosquito net last night? ☐ Yes ☐ No ☐ Don’t know ☐ Refusal

**D4)** Do all household sleeping spaces have a mosquito net? ☐ Yes ☐ No ☐ Don’t know ☐ Refusal

**D5)** Did you purchase them or receive them for free? ☐ Purchase / Number |__|__|; ☐ free/ Number |__|__|

**E.** Health events of the adult chosen by random

E1) Who is he(she)? ☐ The Head of Household (or) ☐ The spouse (go to question E2) ☐ Another adult (complete the chart below)

|  | **Age** | **Sex** | **School level** | **Main activity** | **Marital status *** | **Religion*** | **Nationality** | **Ethnicity** | **Place of birth** |
| --- | --- | --- | --- | --- | --- | --- | --- | --- | --- |
| **Respondent** (e) |  | ☐F ☐M |  | -  - |  |  |  | -  - |  |
| If living in couple, partner |  | ☐F ☐M |  | -  - |  |  |  | -  - |  |

**99 Don’t know, 88 refusal**

**E2)** Did you have a health event the same day of last week = Day1? (Fill in the table- If more than 3 treatments, mark on a sheet)

| **Day 1 (Same day, the last week)** | | | | | | | | | |
| --- | --- | --- | --- | --- | --- | --- | --- | --- | --- |
| **Health Problem:** | | | | **Cause:** | | | **Action1:**  ☐ **Self-medication/** ☐ **Prescribed** | | |
| **Treatment name** | **Type of treatment** | **Presentation** | **Place of supply *** | Adviced By ***** | Prescriber ***** | **Price** | **Dosage** | **Evolution*** | **Action 2:** |
|  |  |  |  |  |  |  |  |  |  |
|  |  |  |  |  |  |  |  |  |  |
|  |  |  |  |  |  |  |  |  |  |
|  |  |  |  |  |  |  |  |  |  |
| **Day 2** | | | | | | | | | |
| **Health Problem:** | | | | **Cause:** | | | **Action1:**  ☐ **Self-medication/** ☐ **Prescribed** | | |
| **Treatment name** | **Type of treatment** | **Presentation** | **Place of supply *** | Advised By ***** | Prescriber ***** | **Price** | **Dosage** | **Evolution*** | **Action 2:** |
|  |  |  |  |  |  |  |  |  |  |
|  |  |  |  |  |  |  |  |  |  |
|  |  |  |  |  |  |  |  |  |  |
|  |  |  |  |  |  |  |  |  |  |
| **Day 3** | | | | | | | | | |
| **Health Problem:** | | | | **Cause:** | | | **Action1:**  ☐ **Self-medication/** ☐ **Prescribed** | | |
| **Treatment name** | **Type of treatment** | **Presentation** | **Place of supply *** | Advised By ***** | Prescriber ***** | **Price** | **Dosage** | **Evolution*** | **Action 2:** |
|  |  |  |  |  |  |  |  |  |  |
|  |  |  |  |  |  |  |  |  |  |
|  |  |  |  |  |  |  |  |  |  |
|  |  |  |  |  |  |  |  |  |  |
|  |  |  |  |  |  |  |  |  |  |
| **Day 4** | | | | | | | | | |
| **Health Problem:** | | | | **Cause:** | | | **Action1:**  ☐ **Self-medication/** ☐ **Prescribed** | | |
| **Treatment name** | **Type of treatment** | **Presentation** | **Place of supply *** | Advised By ***** | Prescriber ***** | **Price** | **Dosage** | **Evolution*** | **Action 2:** |
|  |  |  |  |  |  |  |  |  |  |
|  |  |  |  |  |  |  |  |  |  |
|  |  |  |  |  |  |  |  |  |  |
|  |  |  |  |  |  |  |  |  |  |
| **Day 5** | | | | | | | | | |
| **Health Problem:** | | | | **Cause:** | | | **Action1:**  ☐ **Self-medication/** ☐ **Prescribed** | | |
| **Treatment name** | **Type of treatment** | **Presentation** | **Place of supply *** | Advised By ***** | Prescriber ***** | **Price** | **Dosage** | **Evolution*** | **Action 2:** |
|  |  |  |  |  |  |  |  |  |  |
|  |  |  |  |  |  |  |  |  |  |
|  |  |  |  |  |  |  |  |  |  |
|  |  |  |  |  |  |  |  |  |  |
| **Day 6** | | | | | | | | | |
| **Health Problem:** | | | | **Cause:** | | | **Action1:**  ☐ **Self-medication/** ☐ **Prescribed** | | |
| **Treatment name** | **Type of treatment** | **Presentation** | **Place of supply *** | Advised By ***** | Prescriber ***** | **Price** | **Dosage** | **Evolution*** | **Action 2:** |
|  |  |  |  |  |  |  |  |  |  |
|  |  |  |  |  |  |  |  |  |  |
|  |  |  |  |  |  |  |  |  |  |
|  |  |  |  |  |  |  |  |  |  |
|  |  |  |  |  |  |  |  |  |  |
| **Day 7** | | | | | | | | | |
| **Health Problem:** | | | | **Cause:** | | | **Action1:**  ☐ **Self-medication/** ☐ **Prescribed** | | |
| **Treatment name** | **Type of treatment** | **Presentation** | **Place of supply *** | Advised By ***** | Prescriber ***** | **Price** | **Dosage** | **Evolution*** | **Action 2:** |
|  |  |  |  |  |  |  |  |  |  |
|  |  |  |  |  |  |  |  |  |  |
|  |  |  |  |  |  |  |  |  |  |
|  |  |  |  |  |  |  |  |  |  |

**3)** *If the person has consulted a health professional within last 7 days, ask for information on the consultation* ☐ refusal

| **Day** | **Where (name of the place)** | **District** | **Type of the facility*** | **Profession** | **Consultation cost** | **Why did you choose this place?** | **Satisfaction with care** | **Means of transport** |
| --- | --- | --- | --- | --- | --- | --- | --- | --- |
|  |  |  |  |  |  |  |  |  |
|  |  |  |  |  |  |  |  |  |

**E4)** *If the person has not had a health event during the last seven days*. Excluding medications taking for chronic diseases, when was your last medication taken?

☐ Don’t know ☐ Refusal

| **Date** | **Name of treatment** | **Form of consumption*** | **Symptoms/ Disease** | **Reason for taking the treatment** | **Prescriber*** | **Advised By *** | **Place of supply*** |
| --- | --- | --- | --- | --- | --- | --- | --- |
|  |  |  |  |  |  |  |  |
|  |  |  |  |  |  |  |  |
|  |  |  |  |  |  |  |  |
|  |  |  |  |  |  |  |  |

**E5) Do you know ACT**? ☐ Yes ☐ No ☐ Don’t know ☐ Refusal;

**E6)** Do you take **ACT** in case of malaria? ☐ Yes ☐ No ☐ Don’t know ☐ Refusal

**E7) If not, why**? **_ _ _ _ __ _ _ _ _ _ _ _ _ _ _ __ _ _ _ _ _ _ __ _ _ _ _ __ _ _ _ _ _ _ _ _ _ _ _ _ _ _ _ _ _ _ _ _ _ _ _ _ _ _ _ _ _ _ _ _ _ _ _ _ _ _ _ _ _**

**_ _ _ _ __ _ __ _ __ _ _ _ _ __ _ _ _ __ _ _ __ _ __ _ _ _ __ _ __ _ __ _ _ __ _ _ __ _ _ __ _ _ __ _ __ _ _ __ _ __ _ __ _ __ _ __ _ _ __ __ _ __ _ _ __ _ _ _ __ _**

**_ _ __ _ _ __ __ __ _ _ __ _ _ _ __ _ __ _ __ _ __ _ _ __ _ __ _ __ _ _ _ _ __ _ _ _ __ _ _ __ _ __ _ __ _ _ _ __ _ _____ _ _ _ _ __ _ _ _ __ ___ _ _ _ __ _ _ __ _**

**F1)** Child Information

**F. Health events of the child chosen by random**

| **Age** | **Sex** | **School level**  *(99=NSP ; 88=RR)* | **Nationality** *(99=NSP ; 88=RR)* | **Ethnicity**  *(99=NSP ; 88=RR)* | **Place of birth**  *(99=NSP ; 88=RR)* |
| --- | --- | --- | --- | --- | --- |
|  | ☐F ☐M |  |  | -  - |  |

**F2)** Did the child have a health event the same day last week = Day1? *(*Complete the following table day by day) ☐ Don’t know ☐ Refusal

| **Day 1 (Same day, the last week)** | | | | | | | | | |
| --- | --- | --- | --- | --- | --- | --- | --- | --- | --- |
| **Health Problem:** | | | | **Cause:** | | | **Action1:**  ☐ **Self-medication/** ☐ **Prescribed** | | |
| **Treatment name** | **Type of treatment** | **Presentation** | **Place of supply *** | Advised By ***** | Prescriber ***** | **Price** | **Dosage** | **Evolution*** | **Action 2:** |
|  |  |  |  |  |  |  |  |  |  |
|  |  |  |  |  |  |  |  |  |  |
|  |  |  |  |  |  |  |  |  |  |
|  |  |  |  |  |  |  |  |  |  |
| **Day 2** | | | | | | | | | |
| **Health Problem:** | | | | **Cause:** | | | **Action1:**  ☐ **Self-medication/** ☐ **Prescribed** | | |
| **Treatment name** | **Type of treatment** | **Presentation** | **Place of supply *** | Advised By ***** | Prescriber ***** | **Price** | **Dosage** | **Evolution*** | **Action 2:** |
|  |  |  |  |  |  |  |  |  |  |
|  |  |  |  |  |  |  |  |  |  |
|  |  |  |  |  |  |  |  |  |  |
|  |  |  |  |  |  |  |  |  |  |
| **Day 3** | | | | | | | | | |
| **Health Problem:** | | | | **Cause:** | | | **Action1:**  ☐ **Self-medication/** ☐ **Prescribed** | | |
| **Treatment name** | **Type of treatment** | **Presentation** | **Place of supply *** | Advised By ***** | Prescriber ***** | **Price** | **Dosage** | **Evolution*** | **Action 2:** |
|  |  |  |  |  |  |  |  |  |  |
|  |  |  |  |  |  |  |  |  |  |
|  |  |  |  |  |  |  |  |  |  |
|  |  |  |  |  |  |  |  |  |  |
| **Day 4** | | | | | | | | | |
| **Health Problem:** | | | | **Cause:** | | | **Action1:**  ☐ **Self-medication/** ☐ **Prescribed** | | |
| **Treatment name** | **Type of treatment** | **Presentation** | **Place of supply *** | Advised By ***** | Prescriber ***** | **Price** | **Dosage** | **Evolution*** | **Action 2:** |
|  |  |  |  |  |  |  |  |  |  |
|  |  |  |  |  |  |  |  |  |  |
|  |  |  |  |  |  |  |  |  |  |
|  |  |  |  |  |  |  |  |  |  |
| **Day 5** | | | | | | | | | |
| **Health Problem:** | | | | **Cause:** | | | **Action1:**  ☐ **Self-medication/** ☐ **Prescribed** | | |
| **Treatment name** | **Type of treatment** | **Presentation** | **Place of supply *** | Advised By ***** | Prescriber ***** | **Price** | **Dosage** | **Evolution*** | **Action 2:** |
|  |  |  |  |  |  |  |  |  |  |
|  |  |  |  |  |  |  |  |  |  |
|  |  |  |  |  |  |  |  |  |  |
|  |  |  |  |  |  |  |  |  |  |
| **Day 6** | | | | | | | | | |
| **Health Problem:** | | | | **Cause:** | | | **Action1:**  ☐ **Self-medication/** ☐ **Prescribed** | | |
| **Treatment name** | **Type of treatment** | **Presentation** | **Place of supply *** | Advised By ***** | Prescriber ***** | **Price** | **Dosage** | **Evolution*** | **Action 2:** |
|  |  |  |  |  |  |  |  |  |  |
|  |  |  |  |  |  |  |  |  |  |
|  |  |  |  |  |  |  |  |  |  |
|  |  |  |  |  |  |  |  |  |  |
| **Day 7** | | | | | | | | | |
| **Health Problem:** | | | | **Cause:** | | | **Action1:**  ☐ **Self-medication/** ☐ **Prescribed** | | |
| **Treatment name** | **Type of treatment** | **Presentation** | **Place of supply *** | Advised By ***** | Prescriber ***** | **Price** | **Dosage** | **Evolution*** | **Action 2:** |
|  |  |  |  |  |  |  |  |  |  |
|  |  |  |  |  |  |  |  |  |  |
|  |  |  |  |  |  |  |  |  |  |
|  |  |  |  |  |  |  |  |  |  |

**F3)** *If the person has consulted a health professional within last 7 days, ask for information on the consultation*  ☐ Don’t know ☐ Refusal

| **Day** | **Where (name of the place)** | **District** | **Type of the structure*** | **Profession** | **Consultation cost** | **Why did you choose this place?** | **Satisfaction with care** | **Means of transport** |
| --- | --- | --- | --- | --- | --- | --- | --- | --- |
|  |  |  |  |  |  |  |  |  |
|  |  |  |  |  |  |  |  |  |
|  |  |  |  |  |  |  |  |  |

**F4)** If the child has not had a health event during the last seven days. Regardless of the medications the child takes for chronic diseases, when was his last medication taken? ☐ Don’t know ☐ Refusal

| **Date** | **treatment** | **Form of consumption*** | **Pattern** | **Symptom/disease** | **Place of supply*** | **Prescriber*** | **Counselor*** |
| --- | --- | --- | --- | --- | --- | --- | --- |
|  |  |  |  |  |  |  |  |
|  |  |  |  |  |  |  |  |
|  |  |  |  |  |  |  |  |
|  |  |  |  |  |  |  |  |

**F5)** Do you know ACT? ☐ Yes ☐ No ☐ Don’t Know ☐ Refusal

**F6)** Do you give ACT to children in case of malaria? ☐ Yes ☐ No ☐ Don’t know ☐ Refusal

**F7) If not, why**? **_ _ _ _ __ _ _ _ _ _ _ _ _ _ _ __ _ _ _ _ _ _ __ _ _ _ _ __ _ _ _ _ _ _ _ _ _ _ _ _ _ _ _ _ _ _ _ _ _ _ _ _ _ _ _ _ _ _ _ _ _ _ _ _ _ _ _ _ _**

**_ _ _ _ __ _ __ _ __ _ _ _ _ __ _ _ _ __ _ _ __ _ __ _ _ _ __ _ __ _ __ _ _ __ _ _ __ _ _ __ _ _ __ _ __ _ _ __ _ __ _ __ _ __ _ __ _ _ __ __ _ __ _ _ __ _ _ _ __ _**

**G. Habits**

**Thank you for all the information. Now we would like to know if you have medication in your home pharmacy?**

G1) Do you have home pharmacy? ☐ Yes ☐ No ☐ Refusal

G2) Can we see the drugs? ☐ Yes ☐ No ☐ Refusal

**If the person can't or refuses to show you the drugs, ask him if it is possible to quote you the medicines that he has in his home pharmacy. If he refuses do not insist.**

| **Name of medicine** | **Form of consumption*** | **Place od supply*** | **Reason for taking the treatment *** | **Name of medicine** | **Form of consumption*** | **Place of supply*** | **Reason for taking the treatment *** |
| --- | --- | --- | --- | --- | --- | --- | --- |
|  |  |  |  |  |  |  |  |
|  |  |  |  |  |  |  |  |
|  |  |  |  |  |  |  |  |

**G3)** Do you have a health insurance? ☐ Yes ☐ No

**G3a)If Yes, which one?**  ☐ NHIS or ☐ Private **G3b)** **if private** which one? _ _ _ _ _ _ _ _ _ _ _ _ _ _ _ _ _ ;

**G3c)** Have you renewed your premium? ☐ Yes ☐ No **G3d)** how much Monthly or Annually : |__|__|__|__|__|__|CFA

**G3e)** For your last health event did you use your health insurance? ☐ Yes ☐ No

**G3f)** If No why?

:_____________________________________________________________________________________________________________________________________________________________________________________________________________________________________________________________________________________________________________________________________________________________________________________

**G4)** participation in “Tontine” ☐ Yes ☐No

**G5)** What is the monthly household income? ☐ < 100.00; ☐ 101.00 -200.00; ☐ 201.000 -500.000; ☐ > 501.000; ☐ Unknown ☐ Refusal

Notes on the conduct of the interview :___________________________________________________________________________________________________________________________________________________________________________________________________________________________________________________________________________________________________________________________________________________________________________________________________________________________________________________ *Time at end of interview : |__|__|h/|__|__| min*
